# Remarks on the Surgical Aspect of Glycosuria, and on Diabetic Gangrene, with the Record of Three Cases in Which Amputation Was Performed for Gangrene in Diabetics under Spinal Anæsthesia

**Published:** 1910-12

**Authors:** Charles A. Morton

**Affiliations:** Professor of Surgery in the University of Bristol; Senior Surgeon to the General Hospital and the Children's Hospital; Consulting Surgeon to the Cossham Memorial Hospital.


### REMARKS ON THE SURGICAL ASPECT OF GLYCOSURIA, AND ON DIABETIC GANGRENE, WITH THE RECORD OF THREE CASES IN WHICH AMPUTATION WAS PERFORMED FOR GANGRENE IN DIABETICS UNDER SPINAL ANÆSTHESIA.

BY

CHARLES A. MORTON, F.R.C.S.

*Professor of Surgery in the University of Bristol;*
*Senior Surgeon to the General Hospital and the Children's Hospital;*
*Consulting Surgeon to the Cossham Memorial Hospital.*

THE first question to be considered is what constitutes diabetes? If we say diabetes may be defined as persistent glycosuria, we must remember that the sugar may disappear from the urine for a time in a case in which it has been present for a lengthy period, and in which there have been symptoms commonly present in diabetes. We can hardly regard as diabetes a glycosuria which so far as we know is only temporary, and not accompanied by excessive thirst or polyuria; but on the other hand, we cannot say, because sugar appears in the urine for the first time whilst the patient is under our observation, that it is only temporary diabetes, for the glycosuria may really have only been temporarily absent. In such a case if we have marked thirst, polyuria and wasting, it should certainly make us suspicious that true diabetes was present.

It has long been recognised that temporary glycosuria may follow injury of the central nervous system, but it is believed[1] that glycosuria may also be due to an injury in a limb, such as a fracture, or to a septic disease, such as suppurative appendicitis or pyosalpinx; and Mr. Llewellyn Phillips has recorded a case of spreading traumatic gangrene in which glycosuria appeared during the course of the disease and disappeared after recovery. Moreover, there are at least records of two cases, collected by Mr. Phillips, in which the presence of large abdominal tumours

[1] Phillips, *Lancet*, 1902, i, 1308, and Kausch, *Brit. M. J.*, 1904, ii, 1534.

was associated with glycosuria, and the latter was cured by their removal. If, therefore, we find sugar in the urine of a patient suffering from a fractured leg, or some septic disease, it does not follow that he has diabetes (and in some cases polyuria and marked thirst have been present in this so-called " traumatic glycosuria "). If there is evidence that sugar was not present in the urine before the injury or onset of the disease, or in its early stage, and if it is no longer present when recovery takes place, and does not recur after an interval, without diabetic diet, then we may apparently conclude it was " traumatic glycosuria." But until we had evidence of its permanent disappearance after recovery we could not tell, for, as I have pointed out, its onset during the time the patient is under our observation does not prove it is not true diabetes, for it may have been only temporarily absent. It is very important to recognise that it may apparently be caused by the presence of large abdominal tumours, for the presence of glycosuria in such cases might otherwise be regarded as a contra-indication to operation.

I do not propose in this paper to discuss the desirability of performing various operations in diabetics ; patients often stand such operations very well. Mr. Mayo Robson has quite lately advocated operations which are likely to cure chronic disease of the pancreas in diabetes, for the diabetes may be due to the pancreatic disease. A very exhaustive paper on the surgical aspects of glycosuria and diabetes was published by Mr. Llewellyn Phillips some years ago, [1] and he has shown that though there is an undue liability to sepsis, and many cases die comatose after operation, yet a considerable number have recovered after major operations, and therefore operations for removal of malignant growths, and for acute conditions dangerous to life, should be performed. In my three cases the patients were not adversely affected by the amputations (which were all through the knee-joint), and the most scrupulous precautions as to antiseptic details, and the use of spinal instead of general anæsthesia, enabled me to avoid both the complications of sepsis and coma.

It will be noted in reading the records of my three cases that in

1 Phillips, *loc. cit.* See also paper by Noble, *Amer. Med.*, 1903, vi, 511.

one the sugar had much decreased by diabetic diet, and in another had disappeared with special diet ; but in the third the man had so much pain, and the gangrenous parts were so fetid, I did not like to delay operation for the patient to be dieted. It is probable he had been dieted before admission, but I have no note as to this. It is certainly wise, if we can give the time to it, to diet the patient before operation.

Not only have we to consider the definition of diabetes, but also the question as to what constitutes diabetic gangrene. Every medical man is familiar with the term "diabetic gangrene," and in every text-book of surgery diabetes is given as a cause of gangrene ; but the exact causation of the gangrene is not very fully considered. What is the connection between the glycosuria and the gangrene ? Some surgeons consider [1] that it is due to endarteritis and sclerosis of the arteries, or to nerve degeneration, so that the innervation of the tissues suffers. Does the mere presence of some abnormal chemical constituent in the tissues lead to such failure in their nutrition that they die, and if so why do certain parts only perish ? The tissues, we know, are less able to resist the growth of pyogenic organisms than in a healthy person. Does such a substance act as an exciting cause of proliferative endarteritis, and by leading to the narrowing of the vessel wall produce gangrene in the same way that atheroma does ? In connection with the view that gangrene in some cases of diabetes is the result of failure in innervation of the tissues, Case No. 3 is of interest. There was a perforating ulcer, such as we see now and then in tabes, associated with a spreading, moist gangrene ; but it does not seem likely to me that defective innervation alone would account for both conditions. In some cases the gangrene may be of the "senile" type occurring in a diabetic, *i.e.* there may be blocking of the arteries from atheroma. It would perhaps be best to call such a case senile gangrene occurring in a diabetic. It is said [2] that even if the gangrene be due to some condition in no way dependent on the glycosuria,

[1] Phillips, *loc. cit.*, also article on "Gangrene," in Cheyne and Burghard's *Manual of Surgical Treatment*, vol. i, 1899, p. 63.

[2] Cheyne and Burghard, *op. cit.*

such as ordinary atheromatous obstruction of the lumen of the artery, yet if it occurs in a diabetic, it spreads with greater rapidity, is associated with marked manifestation of inflammation, and is generally of the moist rather than of the dry variety, which is the common form in ordinary "senile" gangrene. In one of my own cases, though glycosuria was persistent, the gangrene was of the dry form, and due to atheroma blocking the vessels It was, in fact, a case of so-called "senile" gangrene occurring in a diabetic.

The prognosis of gangrene in diabetes in bad. Mr. Llewellyn Phillips,[1] writing in 1902, found that during the years 1884–99 there were eleven cases in St. Bartholomew's Hospital not treated by amputation, and they all died; and six out of eight cases treated by amputation died. It would be instructive to know the exact cause of death in all these cases—those operated on and those not operated on—but this Mr. Phillips has not told us. But he has told us that sloughing of the flaps occurred in four of the six cases fatal after amputation. The great probability is that death occurred in most of the cases either from septicæmia or coma. In diabetes the tissues are in a particularly favourable condition for the growth of pyogenic organisms, and the patient is always liable to coma. This coma may come on, of course, after an amputation, and the amputation may therefore be regarded as a determining cause, but I do not know that there are any statistics that would prove that an operation is really a determining factor. It has been thought that the administration of an anæsthetic might produce it, and seeing the close relation between the pathology of diabetic coma and late chloroform poisoning, such a supposition seems quite reasonable. The coma may be a continuation of the anæsthetic coma, or there may be an interval of days. It seems to me that in any operation on a diabetic it would be advisable to avoid a general anæsthetic if possible, certainly chloroform,[2] because most cases of late anæsthetic poisoning have been after chloroform, and also ether, for there have been a few cases of late anæsthetic poisoning after ether. The

[1] *Loc. cit.*

[2] This is the opinion also of Professor Kausch of Breslau, *loc. cit.*

examples which I now record seem to me to show how suitable spinal anæsthesia is for these cases.

Of course, in every operation the most scrupulous care must be taken with regard to antiseptic precaution; operating on these cases is very much like operating on a knee-joint, for the result of carelessness might be most disastrous. In diabetes the tissues seem to have no power to resist the invasion of pyogenic organisms, and even if so few gained entrance into the wound that they would probably be destroyed by healthy tissues, they would most likely, in the tissues of a diabetic patient, lead to suppuration, which might ultimately be fatal, through a general infection.

In all these three cases the form of amputation was the same—Stephen Smith's amputation at the knee joint, by means of lateral flaps. This has always seemed to me a very satisfactory method, as the skin which before covered the knee forms a very suitable covering for the femoral condyles, on which it ought to be possible to bear weight.

In diabetic gangrene, as in "senile," it is generally considered that amputation should be at or above the level of the knee-joint. If diabetic gangrene is from the same cause as is the so-called "senile" gangrene, *i.e.* from blocking of the artery (but in diabetes due to a form of proliferative endarteritis other than ordinary atheroma), clearly we must amputate considerably above the area of the gangrene so as to get above the blocked vessel, and it seems to me that unless we have evidence of pulsation in the tibials at the ankle, we must conclude that the arteries are blocked in the leg, though pulsation in them is not always obtained at the ankle in an apparently healthy limb. If we have a pulsating posterior tibial at the inner ankle, or a pulsating dorsalis pedis, then, even in diabetic gangrene, we might amputate quite near to the gangrenous area.

We ought to consider carefully in what cases of diabetic gangrene amputation is called for. If the area is small, and the gangrene is not spreading, I see no reason why amputation should be done, unless the process of separation of the dead tissue is so tedious that the patient desires to have an operation which may hasten matters. If so, a thorough removal of sloughs may suffice; but if the gangrene is spreading, or is associated with great pain in the living margin of tissue, or there is pyrexia due to septic absorption from the gangrenous area, then the question of amputation must be seriously considered. If the most scrupulous care is taken as to antiseptic details, and spinal anæsthesia used, I do not think the risk of amputation would be great.

**Case 1.**—*A case of dry gangrene due to atheroma of the arteries associated with diabetes.*—E. S. C., aged 66, was admitted to the General Hospital in October, 1907, with dry gangrene of the great toe. The necrotic area in part separated, and was in part removed, but the gangrene spread to the next toes, and the neighbouring part of the foot. No pulsation could be felt in either the posterior tibial or the dorsalis pedis artery in either foot, and the radials were thickened. The popliteal arteries could be felt pulsating on both sides (he was very thin). He had glycosuria on admission, and it persisted during the six months he was in the hospital, but considerably decreased on diabetic diet.

On April 24th, 1908, amputation was performed through the knee-joint under spinal anæsthesia, which acted well. The popliteal artery was very rigid and thick, and the arteries of the amputated leg were found to be extensively affected by atheroma. The lumen of the posterior tibial was almost completely blocked, and that of the anterior was very small. Primary union occurred except at one minute point and the patient was in no way the worse for the operation.

**Case 2.** *Moist gangrene with very thick arteries, probably atheromatous.*—F. M. was sent to me on May 14th, 1907, by Dr. Mackenzie at the General Hospital. Gangrene had been present for three months, and there had been much pain. There was an abundant deposit of sugar in the urine. The arteries could not be felt in either foot, and they were much thickened in the arm. The popliteals could hardly be felt. There was stinking moist gangrene of all the toes, of an inch of the foot close to them, and a large patch of gangrene extended down the sole almost to the heel. On the 15th amputation was performed through the knee-joint under spinal anæsthesia. His general condition was none the worse for the operation, and the wound did not suppurate, but a small portion of one of the flaps sloughed. He was able to leave the hospital in the summer, but Dr. Mackenzie reported his death, I think, in September, from diabetic coma.

**Case 3.** *Spreading moist gangrene, associated with perforating ulcers.*—S. H., aged 57, was admitted into the General Hospital in June, 1908, with a recent patch of gangrene in the sole of the foot, involving the skin and deep fascia, and pus was burrowing more deeply. There was a perforating ulcer on the same foot of six months' duration. The sloughs were cut away soon after admission, but fresh gangrene appeared, and in August the whole foot became greatly swollen, and dusky in patches, with areas of suppuration near the ankle. There was then moderate pyrexia, and his pulse was getting rapid. In June there was sugar in his urine, but this disappeared with diabetic diet. There was no indication of arterial thickening.

On August 13th I amputated through the knee-joint, under spinal anæsthesia. He was none the worse for the operation, and there was primary union of the wound. When seen in October there was a very small dry superficial slough on the skin of the stump.

